# Supplementary material for: Genome-Wide Identification of bZIP Transcription Factors in Faba Bean Based on Transcriptome Analysis and Investigation of Their Function in Drought Response
Source: Plants (Basel). 2023 Aug 24;12(17):3041. doi: 10.3390/plants12173041 (PMC10490193; doi:10.3390/plants12173041)
Supplement: Supplementary file 1 [file plants-12-03041-s001.zip › Supplementary Figure S1.pdf]

| Motif | Legend | Length (aa) | Consensus Sequences |
|---|---|---|---|
| Motif 1 | | 43 | PKRAKRIWANRESAARSRERKQAYIEELERKVQTLQTENTTLK |
| Motif 2 | | 50 | AQLTLYQRDTTGLSNENSELKLRLQAMEQQAHLRDALNEALKKEVQRLKV |
| Motif 3 | | 16 | KKAMPPDKLAECWVID |
| Motif 4 | | 50 | GHSGHRNIQFPQFGHSPSNMPSHQLQQANSHQFSEMLQNDHIGHFKGLDI |
| Motif 5 | | 18 | EDDLFSTYIDLDKLGGCN |
| Motif 6 | | 15 | PRTRHRHSCSMDGST |
| Motif 7 | | 17 | YVDDVWREIQGGDKKWC |
| Motif 8 | | 29 | HHRRAHSEINYRLPDDMMDLSPSDPFNGG |
| Motif 9 | | 17 | MYPHPCMVHMPYPWMPC |
| Motif 10 | | 26 | ATGDIMNHTESYNLGMHPMPFAGSNF |

**Supplementary Figure S1 |** The sequences and lengths of 18 VfbZIP proteins' conserved motifs.
