# Supplementary material for: Genome-Wide Identification of bZIP Transcription Factors in Faba Bean Based on Transcriptome Analysis and Investigation of Their Function in Drought Response
Source: Plants (Basel). 2023 Aug 24;12(17):3041. doi: 10.3390/plants12173041 (PMC10490193; doi:10.3390/plants12173041)
Supplement: Supplementary file 1 [file plants-12-03041-s001.zip › Supplementary Figure S2.pdf]

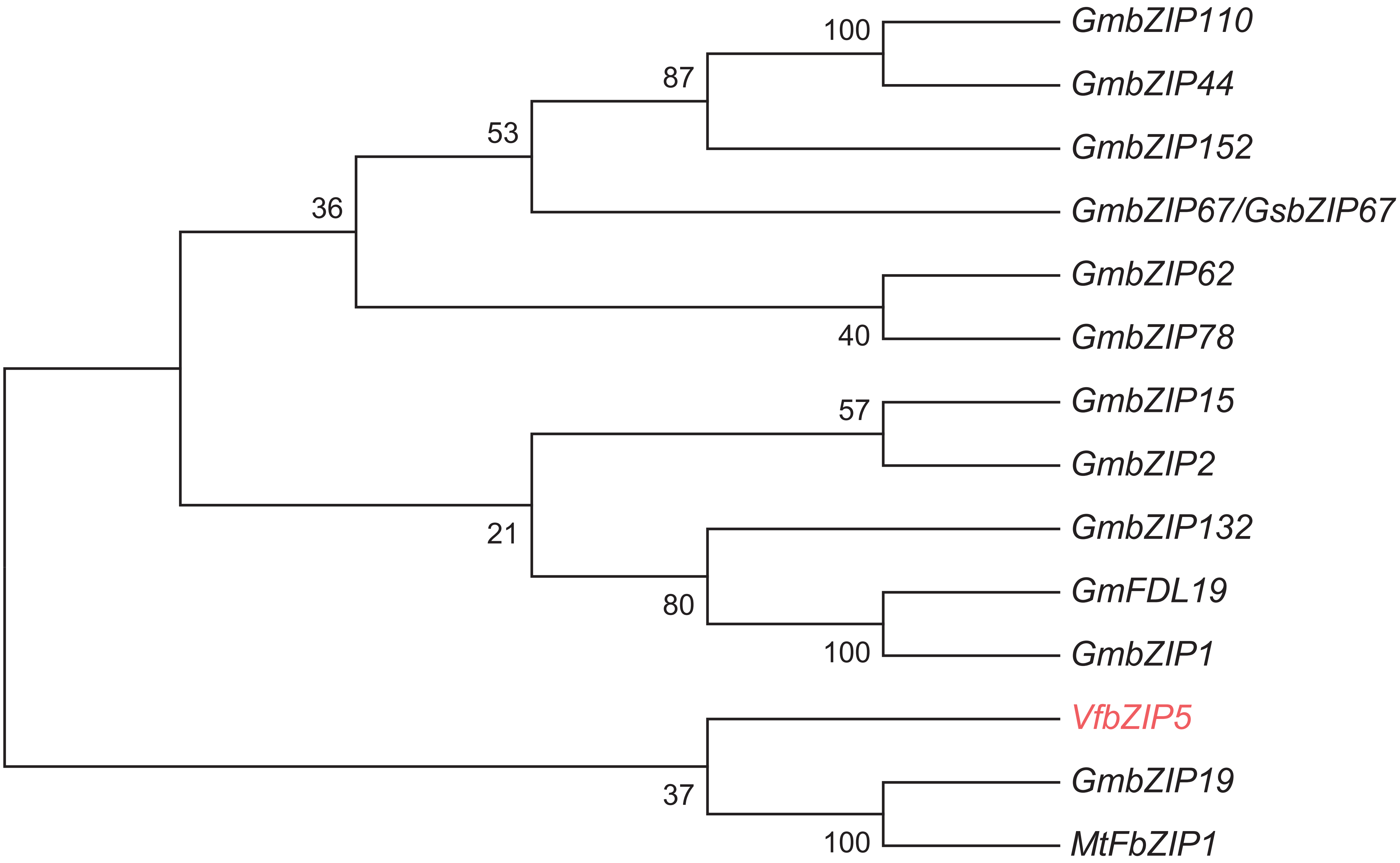

Supplementary Figure S2 | Rectangular phylogenetic tree for comparative analysis of VfbZIP5 with those of other legumes.
